# A Word for Daudet

**Published:** 1889-12-14

**Authors:** 


### A Word for Daudet.

"F." writes: "I was surprised to see in a journal usually so fair-minded as The Hospital an attack on that admirable novelist, Alphonse Daudet. Whether as a philosopher he can be summed up in a Carlylesque phrase—am I wrong in surmising that the two words meant are 'mostly fools?'—I do not pretend to say; I have always taken him simply as a practitioner of the noble art of fiction. He is a storyteller and an artist, cunning in the use of words, conscious of the harmony of their cadences and the subtle impressions conveyed by sound as well as meaning. He can weave a plot, ingenious, but not too complicated; but, above all, he can make his characters live before you. He is not, indeed, a French Dickens, in spite of the Parisian Crummleses who appear in *Fromont and Risler*. If one must compare a Frenchman with an Englishman, I would rather say that the author of the *Nabob*, with his quick eye for social shams, resembles Thackeray. But Daudet is not an Englishman; he is a Frenchman, and his novels are French. He has written *Numa Roumestan*; he has not written *Robert Elsmere*. He has, indeed, given the world one novel with a purpose—*Sappho*; and the purpose has failed conspicuously. Daudet stooped to folly when he imagined that the perusal of *Sappho* would have a wholesome effect on the mind of a young man of twenty, and therefore dedicated the book to his son when he should have reached that age. He is, perhaps, equally unwise in hoping to do good by publishing *La Lutte pour la Vie*; but such unwisdom hardly justifies his being so harshly dealt with as he was in The Hospital.

"In brief the position is this: There is, in France headed by Zola, and in England headed by the sham Zola (Mr. George Moore), a school of novelists who have put the laws of evolution and heredity to the basest uses; have, as Zola himself says, tried to pierce through the evils of circumstance and civilisation in order to find the brute within the soul of man; have clung too fiercely to the doctrine that the evil that men do lives after them. Fiction records life, but it influences it as well; and these novels have, in Daudet's opinion, had an injurious effect on his fellow-countrymen. We, in England, might say the same. My life has not been very adventurous, but I have more than once heard the plea of heredity used to extenuate follies and sins. 'That rash temper which my mother gave me' is a convenient excuse for many things. And surely more than I have heard the 'struggle for existence' euphemised into 'force of circumstances,' 'trade competition,' or some such phrase, employed to condone meannesses of every kind. In short, laws which in themselves are neither moral nor immoral, which, perhaps, should not be called laws, but only reasonable hypotheses—have been most immorally used to exonerate human beings from the responsibility of their own action. The thing has been done in fiction, it has been done in life; and Daudet, who has the artist's insight, sees the danger to which the notion may lead; therefore, by way of warning, he paints in his unlovely colours the man who makes the struggle for existence the excuse for all his selfish cruelty, and says, 'Here he is; behold your *struggle for lifeur*.' The name may be neither good French nor good English, but it clearly defines the monstrous growth of humanity, the false prophet of science whom he has in his eye.

"*La Lutte pour la Vie* may not be such pleasant reading as the delightful adventures of *Tartarin des Alpes*; it may not contain a single phrase that will linger in the memory and the heart like that definition of women like Madame Roumestan—'*les vraies meres, celles qui le sont d'avance*,' but it may be good art all the same. Nay, in pointing out a possible danger in our boasted pride of science, it may be good philosophy too. Not that science itself is dangerous, till men moralise and demoralise it to suit their own ends. As Captain Cuttle has it: 'The bearings of the observation lays in the application of it.'"

